# A Case of Endogenous Trichosporon Endophthalmitis Treated with Micafungin and Voriconazole

**DOI:** 10.4103/0974-777X.52987

**Published:** 2009

**Authors:** Harpreet Walia, Veronica T Tucci, John N Greene, Jennifer Tordilla-Wadia, Patrick Kelty, Sandeep Walia

**Affiliations:** *University of South Florida College of Medicine, 12901 Bruce B. Downs Blvd., Tampa, Florida 33612, USA*; 1 *H. Lee Moffitt Cancer Center and Research Institute, Professor of Internal Medicine and Interdisciplinary Oncology, University of South Florida College of Medicine, 12902 Magnolia Drive, USA*; 2 *Department of Ophthalmology, University of South Florida College of Medicine, 12901 Bruce B. Downs Blvd., Tampa, Florida 33612, USA*; 3 *University of Texas, Austin TX 78712, USA*

**Keywords:** Endophthalmitis, Micafungin, Voriconazole

## Abstract

Invasive fungal infections are a significant cause of morbidity and mortality. Endogenous fungal endophthalmitis is a rare intraocular infection with potential vision threatening consequences. Our review of the literature revealed only one other case of Trichosporon endophthalmitis. Ocular fungal infections are difficult to eradicate because of the limited availability of systemic and intravitreal therapeutic agents and poor tissue penetration of current antifungals. Along with systemic antifungal agents, vitrectomy and intravitreal amphotericin B have been suggested as optimal treatments for fungal endophthalmitis. Other antifungals such as flucytosine and triazoles have recently received consideration. Although the current antifungal therapy is not highly successful, there remains a significant potential for more successful treatments in the future, based on the current studies. We report a case of endogenous trichosporon endophthalmitis that was successfully treated with micafungin and voriconazole. This combination has not been previously reported as a successful therapy in literature. More targeted research is required to uncover additional efficacious therapies to combat trichosporon.

## INTRODUCTION

Invasive fungal infections are a major cause of morbidity and mortality in immunocompromised patients. Although the initiation of antifungal prophylaxis with potent antifungal therapy has reduced morbidity and mortality, novel fungal opportunistic infections have emerged to become a significant source of concern over the past decade. Fungal endophthalmitis is a rare, but visually significant intraocular infection that is difficult to eradicate. We present a case of endogenous trichosporon endophthalmitis successfully treated with micafungin and voriconazole.

## CASE REPORT

A 78-year-old man with a medical history of Acute Myelogenous Leukemia (AML) in a blast crisis, presented for chemotherapy induction. Following chemotherapy, he remained neutropenic and his recovery was complicated by bronchiolitis obliterans with organizing pneumonia, pansinusitis, and renal insufficiency. After treatment with three separate course of steroids, his immune function gradually improved clinically and he began prophylaxis with Azole and Micafungin. After resolution of the initial respiratory symptoms, he again became short of breath and also developed several new skin lesions and blurred vision in his left eye. A computed tomography scan (CT) of the chest demonstrated multiple nodules in his lungs. Based on pulmonary, cutaneous, and ocular involvement, a disseminated infectious etiology was suspected.

Three consecutive sputum cultures were found to be negative for acid-fast bacilli, and blood cultures returned positive for trichosporon. A skin lesion biopsy then microbiologically confirmed disseminated trichosporonosis.

**Figure 1:**
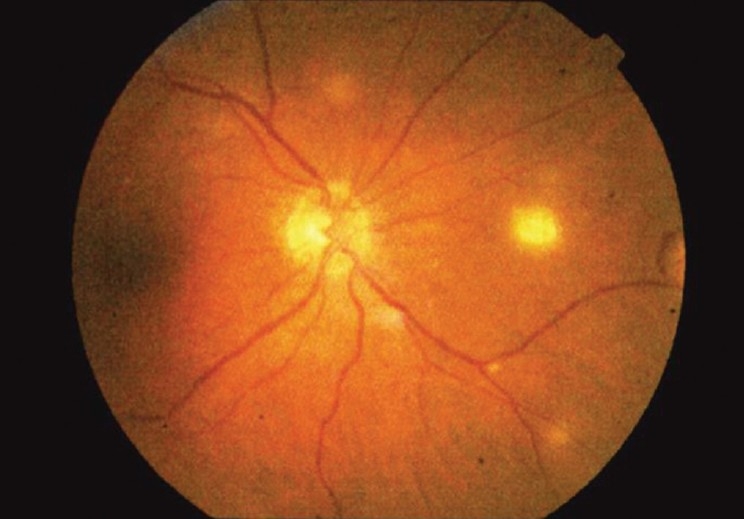
Fundoscopic photo demonstrating chorioretinal lesions near the disc and macula

**Figure 2:**
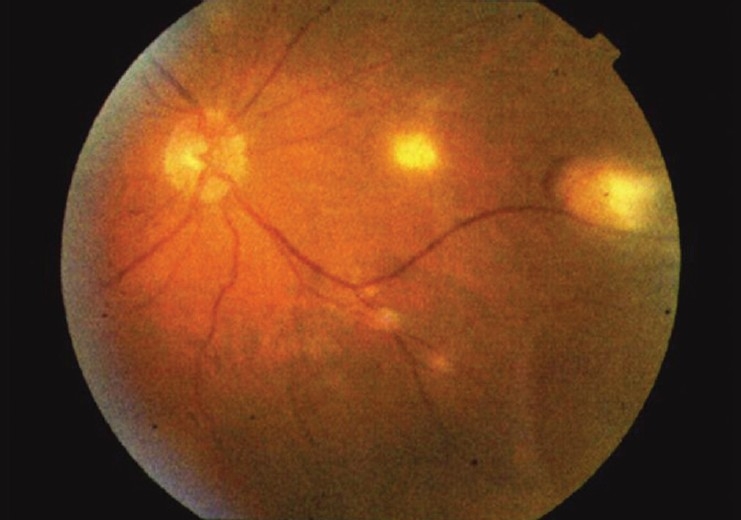
Fundoscopic photo demonstrating multiple chorioretinal lesions consistent with metastatic yeast

**Figure 3:**
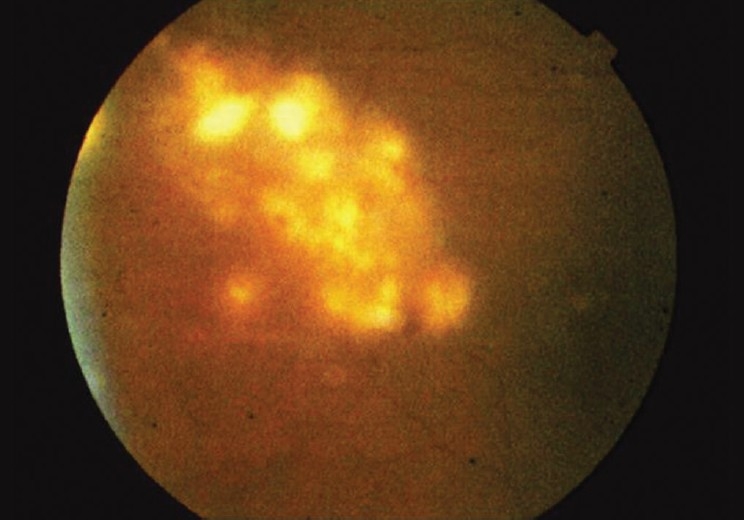
Fundoscopic photo demonstrating multiple chorioretinal lesions in the fundus periphery

The patient complained of blurred vision in his left eye. Best corrected visual acuity was 20/30 in his right eye and 20/40 in his left eye. Visual fields were full to confrontation in both right and left eyes. Extraocular movements were full and no abnormalities were noted on gross examination. Intraocular pressure measured 17 in the right eye and 18 in the left eye. Color vision was within normal limits, as assessed with Ishihara color plates. Slit lamp examination was within normal limits in each eye, both having normal eyelids, white conjunctiva, deep anterior chambers, and round and reactive pupils. There was no sign of anterior segment inflammation or anterior chamber reaction. The patient had posterior chamber intraocular lens in both eyes as a result of cataract surgery in the past. Funduscopic examination revealed multiple chorioretinal lesions in the left eye consistent with metastatic yeast [Figures 1–3]. Based on evidence of disseminated trichosporonosis from blood cultures and skin biopsy, the patient's visual complaints were attributed to trichosporon endophthalmitis.

The patient received a six-week course of micafungin and voriconazole, to help eradicate the disseminated trichosporonosis. His immune function subsequently improved and his visual complaints also resolved.

## DISCUSSION

First reported in 1970, disseminated Trichosporonosis has become increasingly more documented in severely immunocompromised patients.[1] *Trichosporon spp*, formerly known as *Trichosporon beigelii*, includes several genetically distinct species. Trichosporon is ubiquitous yeast that inhabits soil, but may also be normal flora in the human skin and respiratory tract.[1,2] It can cause fatal systemic mycosis in immunocompromised patients, with mortality rates approaching 80%.[1,2]

Entry portals for Trichosporon into deep tissue include the skin and the respiratory and gastrointestinal tracts. The fungus can cause invasive disease in which the lungs, kidneys, skin, and eyes are the main targets. Fungal endophthalmitis is a known complication of disseminated fungal infections occurring after ocular trauma or surgery in immunocompromised patients.[3] The incidence of endogenous fungal endophthalmitis has been reported to vary from 2-45% in patients with systemic fungal infections, with the most common organisms reported including *Candida albicans*, *Aspergillus, Fusarium* species, *Cryptococcus neoformans, Coccidioides immitis, Sporotrichum schenckii, Blastomyces dermatitidis*, and *Histoplasma capsulatum.*[3–5] Trichosporon has only once been reported as a cause of exogenous fungal endophthalmitis, which occurred after cataract extraction in a patient with sarcoidosis.[6] Progression of the infection is influenced by the size and growth rate of the fungus, as well as the state of the host's immune system function.[4]

The diagnosis of trichosporonosis relies on clinical suspicion with microbiological confirmation.[1] Fungal endophthalmitis, although rare among all intraocular infections, is clinically significant due to the vision threatening consequences. Furthermore, ocular fungal infections are traditionally difficult to eradicate because of the limited availability of systemic and intravitreal therapeutic agents and poor tissue penetration of current antifungals.[4] Early detection and prompt diagnosis of fungal endophthalmitis often necessitates vitreous cultures and allows for early antifungal therapy, which gives the best chance at restoring vision and possibly decreasing the morbidity and mortality rate.[3,7] Moreover, the treatment of the systemic fungemia without targeting the local fungal endophthalmitis can result in permanent visual loss.

The majority of reported cases of disseminated Trichosporonosis in immunocompromised patients results in mortality despite antifungal therapy. There is a significant controversy surrounding effective treatment modalities, as *in-vitro* sensitivities do not correlate well with *in-vivo* efficacies. An *in-vitro* study confirmed that azoles are more active *in vitro* than amphotericin B, however, some authors report cases with clinical isolates of *T. beigelii*, which are more susceptible to amphotericin B than azoles.[1,2] Others maintain that antifungal therapy with amphotericin B, fluconazole, and a combination therapy have all been fruitless.[2]

The treatment of fungal endophthalmitis is as controversial as that of systemic Trichosporonosis. Along with systemic antifungal agents, vitrectomy and intravitreal amphotericin B have been suggested as optimal treatments, since amphotericin B has poor intraocular penetration. [6,7] However, the value of this treatment is debatable. Intravitreal amphotericin B has questionable efficacy, while at the same time, its use may be toxic to the retina.[4] Additionally, numerous adverse side effects of amphotericin B may not warrant its routine use in the treatment of fungal endophthalmitis.[4] With increasing resistance to amphotericin B, other antifungals such as flucytosine and azoles can be considered.[7,8] However, flucytosine has a narrow spectrum of activity and ketoconazole has been seen to have poor intravitreal concentration, after oral administration.[4]

Most cases of successful outcomes in immunocompromised patients relate directly to the recovery of the immune function. One case reports that an immunocompromised patient was cured with a combination therapy of miconazole and norfloxacin, and also splenectomy. [8,9] Additionally, pars plana vitrectomy with intraocular debridement can be considered early for optimal preservation of vision.[6] One case reports success with an intracorneal injection combined with intravitreal injection of amphotericin B.[10]

## CONCLUSION

Although it is largely acknowledged that the present antifungal therapy is not very successful in treating endophthalmitis, there remains a significant potential for future treatment with these agents. Investigative studies of triazoles have proved promising as treatment options for disseminated trichosporonosis.[1] Additionally, it has been seen that fluconazole has improved intraocular penetration and voriconazole has a broad spectrum of action *in vitro* against many of the common fungi causing fungal endophthalmitis.[4] Hariprasad has found that oral voriconazole has achieved therapeutic aqueous and vitreous levels in noninflamed eyes and the activity spectrum encompassed the most frequent fungal causes of fungal endophthalmitis.[4] The broad spectrum of antifungal coverage along with good bioavailability after oral administration and limited adverse toxicity makes oral voriconazole an efficient therapeutic agent for the prophylaxis and treatment of fungal endophthalmitis.[4]

These potential options for treating disseminated Trichosporonosis may ultimately eradicate fungal endophthalmitis. Our case is the first in literature to report the successful treatment of endogenous fungal endophthalmitis from trichosporon with micafungin and voriconazole. Additional reports of successful treatment modalities can assist in identifying and refining viable treatment options and regimens in the future.

## References

[1] Erer B, Galimberti M, Lucarelli G, Giardini C, Polchi P, Baronciani D (2000). *Trichosporon beigelii*: A life-threatening pathogen in immunocompromised host. Bone Marrow Transplant.

[2] Paphitou N, Ostrosky-Zeichner L, Paetznick V, Rodriguez J, Chen E, Rex J (2002). In vitro antifungal susceptibilities of Trichosporon species. Antimicrobial Agents Chemother.

[3] Feman S, Nichols J, Chung S, Theobald T (2002). Endophthalmitis in patients with disseminated fungal disease. Trans Am Ophthalmol.

[4] Hariprasad S, Meiler W, Holz E, Gao H, Kim J, Chi J (2004). Determination of vitreous, aqueous, and plasma concentration of orally administered voriconazole in humans. Arch Ophthalmol.

[5] Song A, Dubovy S, Berrocal A, Murray T (2002). Endogenous fungal retinitis in a patient with acute lymphocytic leukemia manifesting as uveitis and optic nerve lesion. Arch Ophthalmol.

[6] Spirn M, Roth D, Yarian D, Green S (2003). Postoperative fungal endophthalmitis caused by *Trichosporon beigelii* resistant to Amphotericin B Retina. J Retinal Vitreous Dis.

[7] Jain A, Egbert P, McCulley T, Blumenkranz M, Moshfeghi D (2007). Endogenous scedosporium endopthalmitis. Arch Ophthalmol.

[8] Jang G, Kim K, Shin W, Lee W (2005). Treatment of *Candida* chorioretinitis with voriconazole. Korean J Ophtalmol.

[9] Ogata K, Tanabe Y, Iwakiri K, Ito T, Yamada T, Dan K (2006). Two cases of disseminated *trichosporon beigelii* infection treated with combination antifungal therapy. Cancer.

[10] Garcia-Valenzuela E, Song C (2005). Intracorneal injection of amphothericin B for recurrent fungal Keratitis and Endophthalmitis. Arch Ophthalmol.

